# Ophthalmology

**Published:** 1897-08

**Authors:** Alvin A. Hubbell

**Affiliations:** Professor of ophthalmology, Niagara University


### Progress in Medical Science.

### OPHTHALMOLOGY.

Conducted by ALVIN A. HUBBELL, M. D.
Professor of ophthalmology, Niagara University.

### OBSTRUCTION OF THE LACHRYMAL DUCT IN NEW-BORN CHILDREN.

LANDOLT (*Annales de Gynéc. et d'Obstét.*, January, 1897,) finds that this condition is not rare and is often overlooked till much harm is done. The affection should be inspected whenever there appears to be conjunctivitis in one eye only a day or two after birth. The conjunctiva itself is not infrequently cured, so to speak, by appropriate lotions. The obstetrician, mistaking a complication or result for a primary disease, finds to his surprise that the eye continues to water, the lids becoming glued together, and a drop of pus often exudes from the inner canthus. This condition is yet more alarming in certain cases where no conjunctivitis has been observed. It looks like the beginning of purulent ophthalmia. Landolt lays down as a rule that obstinate unilateral lachrymation in a newborn child usually signifies obstruction of the tear duct. As an ophthalmic surgeon he advocates sounding of the duct with a fine probe; on no account should the canaliculus be slit up. Afterwards weak antiseptic lotions must be injected into the duct by means of an Auel's syringe; the infant must be turned on its face directly afterwards, lest any of the lotion be swallowed.—*British Medical Journal,* March 6, 1897.

---

### THE GALVANIC CURRENT IN THE TREATMENT OF CORNEAL ULCERS.

PANSIER, (*Annal d' Oculist,* December, 1896,) while employing the galvanic current for its sedative action in inflammations and ulcers of the cornea, has noticed the remarkable effect it has in clearing up the resulting corneal opacities. A current of four or five milliampéres was applied daily for twenty minutes, or even twice a day for fifteen minutes each time, the positive pole being on the neck, the negative on the eyelid. This is continued until the ulcer has cicatrised, or until the scar tissue has become sufficiently transparent. The direct application of one electrode to the eye causes too much pain, even with a current of only two or three milliamperes, to be recommended when there is any corneal inflammation. The usual local treatment of corneal ulcers is carried on at the same time—namely, by galvano-cautery, atropine, and the like.—*British Medical Journal,* February 6, 1897.

---

### OPHTHALMIA NEONATORUM.

CHARTRES (*Arch. Clin. de Bordeaux,* No. 12, December 12, 1896,) has made bacteriological examinations of the pus from twenty-six cases of ophthalmia neonatorum and has found very numerous pathogenic microbes. In 36 per cent. of the patients there were gonococci alone; in other instances there were Löffler's bacilli, 12 per cent.; micrococci, 12 per cent.; streptococci, 8 per cent., gonococci with streptococci, 8 per cent., and in yet other cases there were mixed germs. The worst instances were those in which there were streptococci either alone or associated with gonococci, or the bacilli of Löffler. Such cases ended in loss of vision, or were complicated by a fatally terminating broncho-pneumonia. When the gonococcus alone was found in the pus the prognosis was generally good, treatment resulting in cure. It is easy, therefore, to see that from the point of view of prognosis the bacteriological examination of the discharge at an early stage of the disease is of great importance. The treatment must be energetic from the first, and it ought to be mixed. The author does not deal in this paper with the prevention of the disease, but he remarks parenthetically that while Credé's method is good, care during labor is better still. When the disease is present the first step must be the thorough washing of the eye with a solution of permanganate of potash, so as to reveal the state of the cornea. Chartres then cauterises with nitrate of silver on two or three successive days with frequent eye washings with a solution of boracic acid in the intervals.—(*British Med. Jour.*, February 13, 1897.)

---

### RETROÖCULAR NEURITIS.

Mr. Marcus Gunn, (*British Medical Journal*, March 20, 1897,) in opening a discussion on this subject, characterised retroöcular neuritis as a distinct clinical group of cases and said that, owing to objective signs being not always present, reliance had to be placed on the subjective symptoms ; hence the affection was liable to be considered a hysterical one. The action of the pupils, however, was a most important help in the diagnosis, as in hysterical amblyopia the action was unimpaired, while in retroöcular neuritis the pupil was inactive ; or where the contraction on exposure to light was normal it was not maintained on continual exposure. Occasionally, one or both optic papillæ were involved, but the changes were never gross. In papillitis, due to intracranial disease, the visible changes preceded the loss of vision, whereas in retrobulbar neuritis failure of central vision was one of the earliest symptoms, the visible changes in the nerve occurring later.

During the stage of swelling in papillitis the conductivity of the nerve was unimpaired ; it was only during subsidence, when the exudation was shrinking, that the nerve fibers were compressed, whereas in retroöcular neuritis the pressure on the nerve fibers within the pial sheath, especially where enclosed in the bony optic foramen, produced a very early effect, and was recovered from as soon as the pressure was diminished. One of the most prominent symptoms was the presence of acentral scotonea for colors and for light, indicating an affection of the macular fibers. This might be owing to the situation of these fibers in the nerve or to their great functional activity and consequent sensitiveness to pressure ; or it might be due to the fact that the principal lymph current traversed the center of the nerve. The periphery of the visual field was sometimes contracted ; if this were partial it would be a valuable sign in localising the lesion ; in taking the visual field, care should be exercised in using test objects of a standard size. There was pain on movement, a varying amount of amblyopia, or even where visual acuteness was not diminished, there was slowness in reading the test objects, which occasionally seemed to be moving ; vision was worse in bright light, or after exhaustion or want of food. The sensation of movement of objects might be explained by a breaking up of the medullary sheath, leading to imperfect insulation of the axis cylinders, or by alternating activity of exhausted fibers. The local causes of the neuritis were orbital cellulitis, exposure to cold, general septicemia, periostitis of the optic foramen, extending from the adjacent sphenoidal cells, or the cause might be the local manifestation of a general disease, such as syphilis, rheumatism, or gout, the latter having a tendency to recur. Finally, the optic nerves might be affected as part of the central nervous system. Retrobulbar neuritis had also affinities with toxic affections of the nerve.

---

### THE TREATMENT OF LACHRYMAL OBSTRUCTIONS BY STYLES.

MR. R. SHALDERS MILLER (*British Medical Journal*, March 13, 1897,) says that since 1877, when he first became a clinical assistant at Moorfields, it has been his habit to treat all organic strictures of the lachrymal duct—that is, all parts of the outfall from the lower *punctum lachrymale* to the exit in the inferior turbinate sinus of the nose—by means of styles, in most cases permanently retained. Except for temporary wear, they should be made of pure platinum wire, about 25 gauge, and he always makes them himself for each individual case. The shape, direction and length of the nasal duct being variable, this special adaptation is the key to success in the use of styles, which consist of a vertical stem, with a short arm, bent at or about a right angle to the stem. Usually this arm is made double by recurving the wire back to the stem, so that the arm consists of two thicknesses of wire, parallel and quite close together, the recurved part lying vertically beneath the other. In this way the slit in the edge of the lid is just filled up, but no metal is visible unless the lid be everted. Sometimes the furrow produced by slitting up the canaliculus is very shallow ; then the arm of the style is single.

The length of the arm should be such that it completely, but easily fills the slit-up canaliculus ; it must not be too tight. The stem should always be long enough to project through the lower opening of the *ductus ad nasum* in the inferior nasal sinus. The measure can be readily taken by passing a probe. The practical effect of a style in maintaining the potency of the lachrymal exit depends upon the lateral movements of the lower lid, imparted by those long, straight, muscular fibers which take origin at the tendo palpebrarum, and run horizontally outwards to the surface of the molar bone, and are anatomically quite separable from the oval bundles of the orbicularis proper. The tubular styles of the instrument makers are worse than useless; they soon become choked with mucus and dust. Also, in the case of the grooved styles, the necessarily thin edges of the furrow cause irritation and the production of a granulation which stops the way. The stem of a style should not be straight except in the case of a very short duct, but somewhat concave outwards.

The lower opening of the duct may be obstructed by an excessively incurved lower turbinate, but even this is scarcely an excuse for turbinotomy. He has found no difficulty in pushing a probe through the turbinate bone, and a style, made long, follows. Some of his patients have worn a platinum style for eight or more years without its ever having been removed from the moment of its first introduction.

In every case of lachrymal abscess a style ought to be used for a time at least; in most instances it is needed permanently, but a temporary one must be first inserted of a form modified according to the amount of swelling in the part. Such style may be of lead or silver, and several different forms and sizes are sometimes desirable in succession, to suit the changing size and shape of the lid and canthus. He has found it necessary to make an eyed probe of lead, pass it through the duct and anterior nares, and by means of a thread attached pull a fine drainage-tube through, and thus restore a lachrymal passage that had been obliterated by an encysted abscess for sixteen years. A platinum style was afterwards worn with a perfect result.

---

### GUNSHOT WOUND OF THE EYE.

Mr. Brudenell Carter (*British Medical Journal*, January 16, 1897,) described this case: The patient was a boy, aged 14, who received a charge of snipe shot in his face from a gun, the muzzle of which was only three yards away, on December 14, 1895. Most of the charge entered the mouth, and the jaws were much injured, but two pellets passed through the left lower lid and entered the eye, piercing the sclera below the cornea, one just to the nasal side of the vertical meridian, the other on the same horizontal line and four or five millimeters nearer the nose. Mr. Carter saw the case two days after the accident, and after dilating the pupil was able to see that the retina had been wounded. An expectant treatment was pursued and the eye recovered without inflammation or trouble of any sort. By February 4, 1896, all the blood effused within the eye had been absorbed, and it was then seen that the retina and choroid, having been wounded below the optic disc, had disappeared from an extensive region, probably by retraction, leaving the internal surface of the sclera exposed to view, while on the temporal margin of the gap thus occasioned two pellets of shots were lodged and encapsuled, one slightly above the other, producing elevations which were about a millimeter above the general level of the fundus. Central vision was now equal to $\frac{10}{12}$ of the normal, and the gap in the upper part of the field was too remote from the fixing point to occasion any inconvenience.

---

### RECURRENT ORBITO-PALPEBRAL HEMATOMA IN A HEMOPHILIOUS SUBJECT.

DR. VALUDE (*The Medical Week*, February 19, 1897,) says: Spontaneous hematoma of the orbit and especially of the eyelids is very rare, not over seven or eight cases being known in medical literature. The majority of these patients were arteriosclerotic, or women with vicarious menstruation. Professor Panas, in one case in a child, has attributed an orbital hematoma to digestive disturbances and marked dilation of the stomach. Zehender has met with a case in a child in which hemophilia existed.

In a woman whom I have recently had under treatment for hematoma of the upper eyelid, hemophilia is also the cause of the mischief. The patient in fact bled on the slightest provocation, simply rubbing the skin with a rather coarse cloth being sufficient to cause an emission of blood. Moreover, the blood was found on examination by Dr. Chauffard to be very slow in coagulating, no signs of coagulation being observed until a quarter of an hour had elapsed. The organised elements of the blood were both absolutely and relatively normal as to number. The existence of hemophilia was, therefore, unquestionable. This was the condition when the patient, without the slightest traumatism, developed severe pain in the right orbit, which sometimes led to nausea and vomiting. Simultaneously with this supervened swelling of region, a fluctuating bluish projection made its appearance beneath the upper eyelid, near the supero-internal angle of the orbit. Tapping of this collection gave issue to somewhat thick blood, after which the pain ceased. The swelling and ecchymosis of the eyelid in the neighborhood disappeared after the application of a tight bandage.

A month later the same phenomena were reproduced, but in an attenuated form and were dispelled by simple compression. Since then a bluish and somewhat tender swelling has formed from time to time, but disappears within two or three days on application of a tight bandage.

---

### GONORRHEAL OPHTHALMIA—REPORT OF A CASE TREATED WITH ARGONIN.

Frank Trester Smith (*Journal American Med. Asso.*, April 10, 1897,) reports as follows: C. D. R., male, aged 33. First seen November 25, 1896. Two weeks before, right eye became sore, for which he was treated by his family physician. It was extremely painful until two days before, when something seemed to give way and water gushed from between the lids, after which it became easier.

There was an abundant purulent discharge. The cornea was not clear at any point, but had a fleshy appearance and bulged from the general curvature of the eyeball (total staphyloma).

The man lived at some distance in the country and failed to attend regularly, coming only once or twice a week. For two weeks he was treated in the usual way with boracic acid, bichloride and nitrate of silver with very little effect on the discharge, which was abundant from the time first seen. A microscopic examination by Dr. E. C. Anderson, chief of the Microscopic Laboratory of the Chattanooga Medical College, showed the presence of gonococci in abundance. The patient had gonorrhea.

Thinking this a favorable case to test argonin, as the eye was hopelessly lost, a 5 per cent. solution was instilled into the eye. The application caused no pain and a solution was given to the patient for use at home. Four days later he reported that the discharge had almost entirely ceased after the first use of the argonin. He had kept samples of the discharge, which were preserved on cotton swabs made by wrapping pledgets of cotton on toothpicks. These were sealed in envelopes as soon as obtained. An examination of these failed to show a single gonococcus. A sample of the discharge taken direct from the inside of the lid was found to contain a few, very few gonococci.

The argonin was continued. A few days later the eye again became very painful. The tension was increased to plus 1 and the staphyloma was larger. The ball was incised and the clear lens escaped. There was no pus inside the eye. There was no further pain and the patient ceased his visits owing to sickness in his family. One of his neighbors reported that he had no further trouble. The fellow eye was unaffected during the whole of the treatment.

This case seems to indicate that argonin can be used safely in the eye; that it is less irritating than nitrate of silver and from its wonderful effect on the discharge and development of the gonococci it appears to be the ideal remedy in purulent ophthalmia. Further tests will demonstrate its true value.

